# Supplementary material for: Conservation implications of herbicides on seagrasses: sublethal glyphosate exposure decreases fitness in the endangered Zostera capensis
Source: PeerJ. 2022 Nov 15;10:e14295. doi: 10.7717/peerj.14295 (PMC9673766; doi:10.7717/peerj.14295)
Supplement: Supplemental Information 1 — Supplementary Materials for van Wyk et al._impacts of glyphosate on seagrass conservation [file peerj-10-14295-s001.docx]

*Supplementary material*

**1. Experimental design**

Control

250µg/L

750µg/L

2200µg/L

Pot containing 10 shoots

Filter and aerator pump

Figure A.1 Experimental design consisting of 18 water tanks, each containing two pots planted with 10 shoots of *Zostera capensis* each. The water tanks are placed in two connected water baths to equalise temperature and assigned to treatments or control via a randomised block design.

**2.Calculating glyphosate exposure during the experimental period**

**Table A.1** Measured glyphosate concentration in treatment groups at 24 hr.

| **Treatment** | **Control** | **250 µg/L** | **750 µg/L** | **2200 µg/L** | **Tank** |
| --- | --- | --- | --- | --- | --- |
| **Concentration** | 0 | 158 | 369 | 1802 | 1 |
|  | 0 | 144 | 436 | 1530 | 2 |
|  | 0 | 182 | 580 | 1232 | 3 |
|  | 0 | 107 | 749 | 767 | 4 |
| **Mean concentration** | 0 | 148 | 534 | 1333 |  |

**Table A.2** Calculated time weighted average (TWA) concentration of glyphosate in µg/L, over the three-week experimental period, based on nominal concentrations administered. Time weighted average glyphosate concentrations were calculated using a 20% loss per week due to water changes, and assuming no further degradation or adsorption, with the possibility that overall exposure levels were even lower than these calculated values.

| **Treatment** | **Week 1** | **Week2** | **Week 3** | **TWA/group** |
| --- | --- | --- | --- | --- |
| **Control** | 0 | 0 | 0 | 0 |
| **250µg/L** | 250 | 200 | 160 | 203 |
| **750µg/L** | 750 | 600 | 480 | 610 |
| **2200µg/L** | 2200 | 1760 | 1408 | 1789 |

**Table A.3** Calculated time weighted average concentration of glyphosate over the three -week experimental period, based on measured glyphosate concentrations at 24 hr.

| **Treatment** | **Week 1** | **Week2** | **Week 3** | **TWA per tank** | **TWA per group** |
| --- | --- | --- | --- | --- | --- |
| Control | 0 | 0 | 0 | 0 | 0 |
| 250µg/L | 158 | 127 | 101 | 129 | 120 |
| 250µg/L | 144 | 115 | 92 | 117 |  |
| 250µg/L | 182 | 145 | 116 | 148 |  |
| 250µg/L | 107 | 85 | 68 | 87 |  |
| 750µg/L | 369 | 295 | 236 | 300 | 434 |
| 750µg/L | 436 | 349 | 279 | 355 |  |
| 750µg/L | 580 | 464 | 371 | 471 |  |
| 750µg/L | 749 | 599 | 480 | 609 |  |
| 2200µg/L | 1802 | 1441 | 1153 | 1465 | 1084 |
| 2200µg/L | 1530 | 1224 | 979 | 1245 |  |
| 2200µg/L | 1232 | 986 | 789 | 1002 |  |
| 2200µg/L | 767 | 614 | 491 | 624 |  |

**3. Morphological analysis on full dataset**

**3.1 Boxplots of untransformed data**


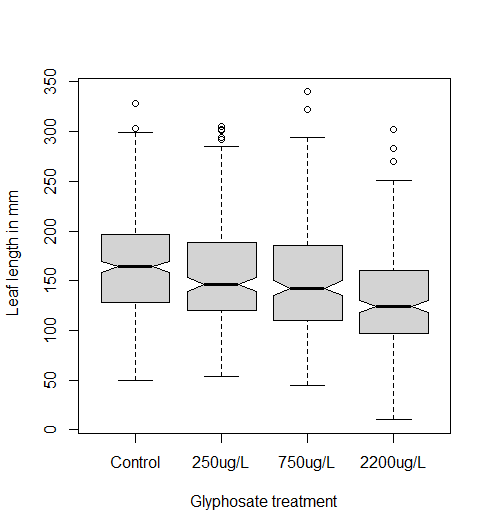


**Figure B.1** Notched boxplot showing medians and interquartile range of untransformed data (full dataset), showing the decrease in leaf length (mm) with increasing glyphosate concentration (µg/L). Notches that do no overlap give a strong indication that medians are significantly different.


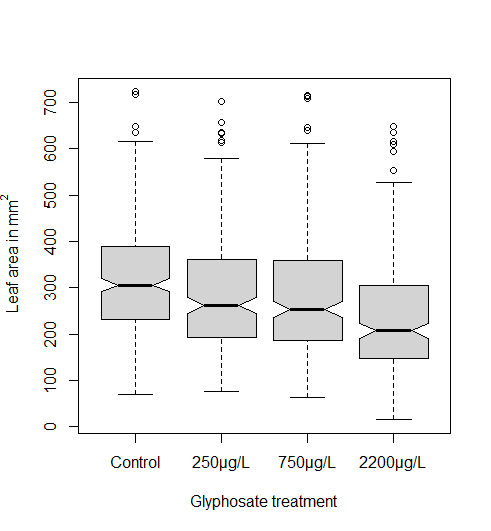
**Figure B.2:** Notched boxplot showing medians and interquartile range of untransformed data (full dataset), showing the decrease in leaf area (mm²) with increasing glyphosate concentration (µg/L ). Notches that do no overlap give a strong indication that medians are significantly different.

**2.2 ANOVA on full morphometric dataset**

Analysis was run on the full dataset, with n equal to 360 for the control group and 240 for the each treatment group. Glyphosate concentration had a strong, statistically significant negative effect on leaf area (F value 29.041**,** p <0.001), although an interaction between water baths and glyphosate treatment was also detected (F value 12.864, p <0.001), as well as a batch effect for bath alone (F value 9.0908, p =0.002). The same was true for leaf length (F value 25.5771, p<0.001), which also had an interaction with water bath (F value =15.0030, p= <0.001) (Type III ANOVA), as well a batch effect for bath alone(F value =7.9118, p= 0.002).

ANOVA was performed on leaf width data. Glyphosate concentration had a strong, statistically significant negative effect on leaf width (F value 27.5642, p<0.001), although an interaction between the two water baths and the glyphosate treatment was also detected (F value 7.7551, p <0.001) (Type III ANOVA) as well a batch effect for bath alone (F value = 7.1952, p= 0.007).

**3. Photosynthetic pigments analysis**

**Table B.1**

| **Calculations used for chlorophyll and carotenoid determination** |
| --- |
| Chlorophyll a (µg/g fw) = 12.25(A663.5)-2.55(A646.5) |
| Chlorophyll b (µg/g fw) = 20.31 (A646.5) – 4.91(663.5) |
| Carotenoids (µg/g fw) = (1000A470 – 3.27[chlorophyll a] – 104[chlorophyll b])/227 |
| Total chlorophyll = 17.76(A646.5)+ 7.34(A663.5) |


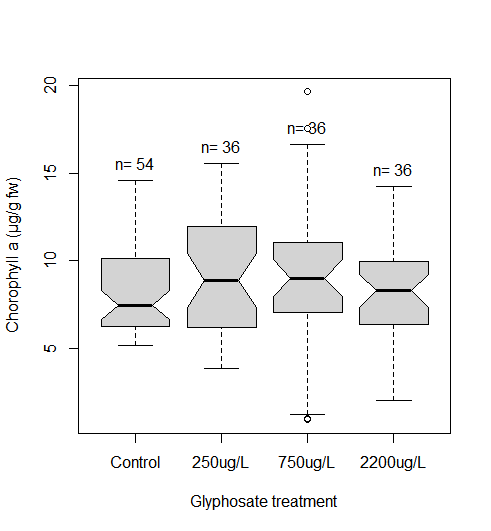

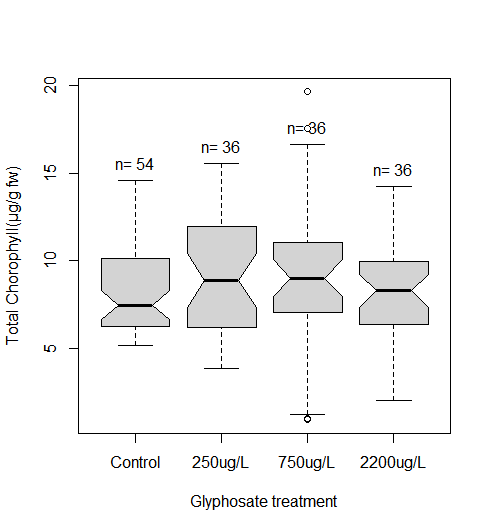
Data did not conform to the requirements for normality, therefore a Kruskal-Wallis rank sum test was performed. The results are given in Table B.2 below, and the median and data spread in Figure C.1.


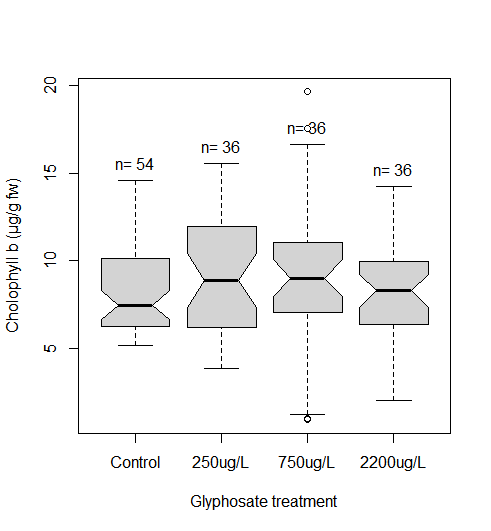
**Figure C.1 Notched boxplots showing effect of glyphosate concentration on total chlorophyll, chlorophyll a and chlorophyll b concentration.**

**Table B.2** Results of Kruskall-Wallis test on photosynthetic pigment concentration

| Pigment | Chi-squared | df | p-value |
| --- | --- | --- | --- |
| Total chlorophyll | 3.464 | 3 | 0.326 |
| Chlorophyll a | 1.344 | 3 | 0.719 |
| Chlorophyll b | 2.77 | 3 | 0.428 |
